# Time to Ban Lead in Industrial Paints and Coatings

**DOI:** 10.3389/fpubh.2015.00144

**Published:** 2015-05-18

**Authors:** Perry Gottesfeld

**Affiliations:** ^1^Occupational Knowledge International, San Francisco, CA, USA

**Keywords:** lead paint, lead poisoning, industrial coatings, REACH, lead exposure

In the U.S. and most high-income countries, regulations already restrict the use of lead paint for residential applications. However, few countries have enacted comprehensive bans on the use of lead additives in all paints. In 2009, more than 120 countries at the UN International Conference on Chemicals Management (ICCM) voted to phase-out all lead paints (1). Since then, a few countries including the Philippines and Nepal have enacted regulations to eliminate the use of lead additives in both consumer and “industrial” paints, but most countries have no restrictions on the manufacture or use of lead in any type of paint (2).

The hazards of lead paint have been known since at least the 1800s and even the recommended alternatives to lead pigments advocated in that era are still used in making paints today (3). Yet, the debate on banning lead paint still rages in capitols from Dhaka to Brussels despite overwhelming evidence that workers and children are harmed from lead exposures resulting from these applications.

In the European Union (EU), a fight is currently under way over a petition to exempt Lead Chromate pigments from the registration, evaluation, authorization, and restriction of chemicals (REACH) regulations. It is an unusual battle that has pitted the world’s largest lead pigment manufacturer, the Canadian-based Dominion Colour Corporation, against some European industry associations and AkzoNobel, the world’s largest paint manufacturer, which eliminated the use of lead in all their products in 2011 (4, 5).

Although in many countries, architectural/decorative paints still contain significant concentrations of lead, “industrial” paints generally have lead concentrations that are up to 10 times greater. For example, road marking paints can contain up to 20,000 ppm lead (6).

However, there are well known substitutes for lead additives in all types of paints and coatings used for all applications. Despite the availability of substitutes, multi-national paint companies often sell lead-free coatings in some markets while they continue to market lead-containing products in jurisdictions where there are no regulatory constraints and customers are less aware of the hazards.

In the U.S., efforts to regulate the lead content of paints initially focused on decorative/architectural paints and consumer products. Subsequently, large paint purchasers in the U.S. had begun to assess the costs of safely maintaining and eventual demolishing industrial structures, bridges, ships, and roadways with lead paint and elected to require lead-free paint and coatings in project and product specifications.

## Concerns with Industrial Applications

But progress in switching to safer non-lead alternatives has been slow in the rest of the world and the lack of awareness of the problem is certainly one reason. The continuing manufacture and use of paint containing lead for “industrial” applications poses substantial health concerns. These concerns include

- There is no regulation or universal definition to differentiate “industrial” coatings from “architectural/decorative” coatings. Unless a regulation restricts all uses of lead additives in paints then there is no realistic way to ensure that “industrial” coatings will not be used in homes, schools, or hospitals.
- Furniture, toys, and other consumer products can be coated with “industrial” paints because even today these applications are not regulated in most countries and they remain a continuing source of childhood lead exposure.
- In developing countries, many small businesses are located in and around housing. The application or removal of lead paint in automotive repair and in the production of crafts and other goods can be a source of exposure to children and others residing in the vicinity.
- Workers are exposed to hazardous levels of lead in the manufacture of lead paint and in the application and removal process. These workers often bring the lead home on their clothing and bodies in the form of contaminated dust and expose their families. This is another common source of lead poisoning among children.
- The use of lead paints and coatings on steel structures, road markings, and in consumer products (e.g., automobiles) is a significant source of environmental contamination. Lead in soil from routine weathering of exterior paints, or from routine maintenance, repainting, and demolition of steel structures, is a common source of childhood lead poisoning.

Public health experts have urged elimination of lead in all paints and coatings. Just this year, the International Society of Environmental Epidemiology called for a ban (7).

## Hazards of Lead Exposure

The World Health Organization (WHO) estimates that 240 million children are over exposed to lead above the reference level established by US CDC of 5 μg/dl of lead in blood (8). Low level lead exposures account for 674,000 deaths per year, primarily due to its contribution to cardiovascular disease (9).

In 2012, the U.S. National Toxicology Program (NTP) conducted a thorough review of the health effects of low level exposures to lead and concluded that “there is *sufficient* evidence that blood lead levels <5 μg/dl in children are associated with increased diagnosis of attention-related behavioral problems, greater incidence of problem behaviors, and decreased cognitive performance” (10). In adults, they found that these same levels were associated with reduced kidney function and that levels <10 μg/dl are associated with neurocognitive decline. Noting that there are more than 28,900 publications on the health effects of lead, the NTP report emphasized the strength of the science in reaching these conclusions.

Public health officials have repeatedly warned that that there is no known “safe” level of lead exposure. As a result, in 2012, the Advisory Committee on Childhood Lead Poisoning Prevention (ACCLPP) to the CDC recommended the discontinuation of the designated blood “level of concern” and instead prioritized the most highly exposed children based on the current reference value of 5 μg/dl (11). The lack of any evidence of a threshold for harm from lead exposure was also noted by WHO (12).

The ACCLPP report indicated that since the health effects of lead appear to be irreversible in the absence of any other interventions, public health policies should encourage the prevention of lead exposures (11). Lead additives in both decorative and “industrial” paints/coatings can contaminate the environment and are a known source of lead poisoning in both children and adults.

## Children and Workers are Exposed

Lead in “industrial” paints/coatings expose workers during manufacturing, application, maintenance, repainting, and eventual removal and/or demolition. Children and others in surrounding communities are exposed to airborne lead released during paint removal. Regular maintenance of metal structures requires that the lead paint be periodically removed down to the substrate. Soil and dust contamination resulting from these operations also results in exposures to children. Containment of these steel structures during the removal of lead paint is costly and generally results in higher exposures to workers on the interior of the containment barriers.

Lead poisoning cases from exposures to almost every type of “industrial” application of lead paint have been documented. Below is a summary of some examples of lead poisoning cases linked to manufacturing and use of these coatings.

### Bridges

Studies conducted during paint removal with abrasive blasting on bridges have documented significant exposures. For example, one study conducted in Chicago, Illinois during abrasive blasting showed worker exposures exceeded the Occupational Safety and Health Administration (OSHA) permissible exposure limit (PEL) of 50 μg/m^3^ by 219 times (13). In Holland, airborne exposures to lead during the demolition of a railway bridge coated in a lead primer were as high as 38,000 μg/m^3^ or approximately 760 times the PEL (14). Air monitoring done during surface preparation for the repainting of a highway bridge in Massachusetts indicated that 18% of samples taken more than 6 feet from the exterior of the containment exceeded the PEL (15). Eighty percent of workers’ exposures on this job exceeded the OSHA PEL.

### Marine

Geometric mean airborne lead exposures during sanding of lead paint on ship overalls in a Navy shipyard were 61.0 μg/m^3^, exceeding the OSHA PEL by 21% (16). Elevated airborne exposures and occupational lead poisoning are common in ship breaking activities. In Thailand, a study demonstrated extensive soil and household dust contamination decreasing inversely with the distance from boat repair yards and provided evidence of take home exposures leading to dust contamination in worker’s homes (17).

### Automotive

Lead paints are a hazard to workers applying these coatings as well as to workers in automotive repair. For example, a study of automotive repair shops in Rhode Island found elevated blood lead levels among workers involved in painting operations and concluded that “vehicle paint dust present in the occupational environment is the principal source of lead exposure” (18). Workers involved in spray-applied lead paint during auto repair in Thailand had blood lead levels approximately two fold greater than controls (19).

### Manufacturing lead paint

Researchers found that workers in a Kenyan paint factory were subjected to average airborne exposures to lead that significantly exceeded the U.S. OSHA PEL (20). The authors of the study also reported that workers’ blood lead levels in the paint factory were more than three times higher than the level triggering notification as a medical condition in the U.S. The study indicated that 75% of the paint manufacturing workers had blood lead levels that exceeded 30 μg/dl.

## Recommendations

As noted, some countries (e.g., Nepal and the Philippines) have enacted regulations restricting the concentration allowed in “industrial” and decorative paints. Others have placed restrictions on specific lead compounds used as pigments or driers in paints. Perhaps Australia has the most comprehensive list of lead compounds that have been banned for use in paints since 2008 with some exceptions (21).

The EU has restricted the use of some specific pigments for residential applications and recently initiated action to ban the use of lead chromate pigments for additional applications. However, the REACH process allows companies to apply for exemptions (i.e., authorizations) to these restrictions, and in the case of lead chromate pigments, have accepted false assertions that alternatives are not available. It is more difficult and costly to verify compliance with these kinds of chemical-specific restrictions rather than outright limits in the lead concentration allowed in paints. That has been the approach in the U.S. and Canada where there are restrictions on the total lead content of paints at 90 ppm, without regards to specific pigments or drier additives.

Although, the U.S. has no restrictions on the lead content of industrial paints, this has partially been addressed by prohibitions on the part of federal and state governments for public works projects. Over 20 years ago, the U.S. Department of Transportation conducted extensive-independent testing of non-lead alternatives for steel bridges and concluded that these substitutes “are currently widely used in new construction due to their excellent long-term corrosion control performance” (22). Therefore, state and local highway and transport agencies have generally prohibited the use of lead paint for road markings, bridges, and other steel structures.

It is now time for the U.S. to take action to expand existing prohibitions on the use of lead paint to include “industrial” paints and coatings. Currently, only the U.S. Consumer Product Safety Commission (CPSC) has regulations restricting the use of lead paint in specific consumer products. However, since there are well-documented environmental and health impacts from the continued use of lead paint on ships, cars, steel structures, bridges, roadway markings, and other applications, further restrictions would require the Environmental Protection Agency to initiate rulemaking.

In addition to protecting the environment at home, U.S. efforts to restrict or ban “industrial” paints would help promote lead paint elimination efforts globally. The lack of regulation in the U.S. has become an impediment for some countries to adopt laws and regulations to eliminate lead in paint.

The available evidence suggests, and written comments from paint industry leaders acknowledge, that paint manufacturers already have access to the available substitutes to eliminate lead from paint in all applications. These substitutes for lead pigments and driers are available globally and have been demonstrated to perform equally or better over time.

In the past two decades, almost all countries have put in regulations to ban the use of lead additives in fuels. The success of this effort must now be replicated in banning the use of lead in paints and coatings.

Now is the time for a concerted effort to expand existing restrictions to finally ban these dangerous and unnecessary uses of lead compounds. This would most efficiently be accomplished by regulating the total lead content of paints and coatings for all applications rather than the piece meal approach that invites a fight on each compound.

## Conflict of Interest Statement

The author declares that the research was conducted in the absence of any commercial or financial relationships that could be construed as a potential conflict of interest.
